# Notes of the Week

**Published:** 1921-04-02

**Authors:** 


### NOTES OF THE WEEK.

### WHAT WILL THE APPROVED SOCIETIES DO?

The Minister of Health has lost no time in circulating the interim report of Lord Cave's Committee amongst the Approved Societies. In so doing Dr. Addison sends a covering letter expressing the hope that each Society will give the report careful and friendly consideration before finally arranging any scheme of additional benefits. He also points out that Section 15 of the Act of 1920 is so worded that a Society may, as an additional benefit, either make payments towards the cost of the maintenance and treatment of each individual member who becomes an inmate of a hospital or give periodical subscriptions to hospitals in return for which treatment of members will be secured as may be reasonably commensurate with the amount of the subscription. Where the Minister has given provisional sanction to schemes it is open to a Society to reconsider the hospital bearing and to submit a revised scheme, including provision in this respect. Lord Cave's Committee estimated that 30 per cent. of the out-patients and a larger percentage of the in-patients are insured persons, and this statement is borne out by individual estimates given at annual meetings of hospitals at which, with few exceptions, the insured patient problem takes a leading place in the report of the executive. As an instance it was stated that a capitation grant paid for National Health patients attending the Birmingham and Midland Eye Hospital would have the effect of making the hospital pay its way instead of being burdened by a deficit, such as that of last year. Although the quaint suggestion has been made that the hospitals should withhold any claim until the next valuation of societies in 1923, we have reason to believe that most of the large Approved Societies are in favour of an equitable arrangement, although no definite announcements have been made. It is stated that some of the Societies have already prepared for the payment of a cash benefit in July, but this can easily be altered to meet the circumstances. If the chief societies show a lead we are confident that the smaller ones will come into line.

### BRADFORD MUNICIPAL HOSPITAL.

It appears that a report of the Advisory Medical Committee appointed by the medical profession of Bradford "to deal with the Health Committee on all matters relating to the public health of the city" which found its way into the newspapers in February last was premature. The final report, which apparently is a re-draft of what appeared, has now been published. With regard to the proposals of the Medical Officer of Health (Dr. J. J. Buchan) as to the setting up and administration of St. Luke's Municipal Hospital, the findings of the Medical Committee are that the scheme is one which aims at establishing a municipal hospital of such magnitude that it will lead to the extinction of the existing voluntary institutions, and which contemplates taking over work which is now being adequately performed by the existing voluntary hospitals and the medical practitioners of the city. The Committee estimates the cost of the upkeep of St. Luke's Hospital at £240,000 a year, or a charge of 16s. per head of the population of the city per annum, and puts forward as an alternative a scheme of co-ordination of all the hospitals of Bradford—namely, the three voluntary hospitals and St. Luke's—in such a way that each institution will become one branch of a conjoint hospital scheme. It is proposed that the group of hospitals should be under a joint board with executive powers, comprising representatives of the voluntary hospitals and of the Health Committee (representing St. Luke's Hospital), but that internal affairs shall continue to be administered by each board of management, all matters which affect the hospitals as a conjoint institution being dealt with by the joint board.

### MEDICAL STUDENTS AND BRADFORD.

It was stated at the annual meeting of the Royal Eye and Ear Hospital, Bradford, that in view of the unsettled hospital position in the city, and the fear that shortly all the medical practitioners and consultants will be under municipal control, no students are contemplating the practice of the profession in Bradford. This we think can be but an individual opinion, but we sympathise with the managers of the voluntary hospitals of the city in their difficulty in obtaining funds when everybody is threatened by a substantial increase in the rates for the purpose of the proposed municipal general hospital. But, as we have said before, it is almost impossible to obtain any but extreme partisan views on the situation, and what is wanted is a report by an independent authority. At the annual meeting of the Royal Infirmary it was argued that another hundred general beds are all that is required instead of the 1,150 proposed by the Health Committee. We are glad to learn that the Lord Mayor of Bradford has offered to arrange, if possible, a meeting between representatives of voluntary hospitals and the Health Committee to endeavour to bring about a working arrangement to save the rates from being burdened by unnecessary expenditure.

### PEACE PROGRAMME OF THE RED CROSS.

The humble penny is now sought by most charitable undertakings, and the British Red Cross Society is the latest to take up its quest in an organised manner. Sir Arthur Stanley, explaining the new movement, has stated that an endeavour is to be made to enlist the services of all in the great campaign against disease and suffering. Each branch of the Red Cross throughout Great Britain is now engaged in obtaining recruits, who are asked to pay a penny a week or 4s. 4d. annually, and it is expected that there will be great rivalry among the counties in novel efforts to obtain new members, especially as each county will administer its own funds. The Peace programme of the Red Cross includes the care of wounded ex-Service men, courses of instruction to fit volunteers to attend those suffering at home from tuberculosis, and duties at infant welfare centres. An additional important feature is to lessen the financial strain on hospitals.

### VENEREAL PROPAGANDA.

It is satisfactory to note the extent of the propaganda work already undertaken in connection with venereal disease, and taking one body alone—namely, the National Council for Combating Venereal Diseases—one learns that the medical department has received and answered 33,000 personal letters of inquiry, 606,000 free pamphlets have been individually distributed, and a very large number of addresses have been given to teachers, social organisations, and unselected audiences of the general public. Of the Council's eighty-nine branches, one claims to have reached in this way, during the last three months, 33,000 people.

### CONTRIBUTORS' CERTIFICATES.

The March number of the Journal of the Great Northern Central Hospital in Holloway is naturally chiefly filled with the account of the institution's amalgamation with the Royal Chest Hospital in City Road. The familiar arguments for centralisation, economy, and the advantage of a large hospital as a magnet for public support are tersely presented. Another "innovation" discusses the new system of contributors' certificates, which was found desirable when payments from patients were introduced. These payments led to requests for exemption from past or present contributors, and the certificates are granted to satisfy those "who ask for a return for their gifts." Contributions now entitle donors or collectors with boxes to certificates of "a face value equal to the amount of the gift." Only those unable to pay for private medical attendance and in need of hospital treatment are eligible. The certificates are "worth" half-a-crown, eighteenpence, sixpence, or threepence, and are available for "the bearer" at any time during ten years. On presentation and acceptance the certificates entitle patients to exemption from a payment equivalent to the value of the certificate, and if the latter is less than the payment the difference only has to be paid. The almoner keeps those not required by the donor, and allots them to deserving cases. Those in the private, contributory, or the general wards may benefit by the certificates, and also those in the out-patient and special departments. The scheme, which began on February 3, is not retrospective. Our only criticism of the plan is the implication that contributions are given for services to be received. All contributions to a voluntary hospital are given for the benefit not of the donor but of others, and though patients, like other classes, may be glad to contribute, the commercial basis never is, and never can be, the foundation or motive for any gifts.

### DANGEROUS DRUGS ACT INQUIRY.

The Committee set up by the Home Secretary, to consider the representations of various bodies affected by the Regulations to be made under the Dangerous Drugs Act, took evidence from these organisations last week from medical practitioners, chemists, veterinary surgeons, and representatives of hospitals. The sitting was not open to the Press, so that it is not possible to make public the evidence submitted at present. Judging by the several schemes for hospitals which have been submitted, there appears to be some difference of opinion amongst hospital and institutional pharmacists as to the method of ordering the drugs covered by the Regulations. Some favour allowing the sister-in-charge of a ward to order morphia injection, tablets, etc., for "stock" use, whilst other hospital pharmacists submit that all requisitions ought to be signed by a resident or visiting medical officer. It is understood that the Committee favours a separate set of Regulations for public hospitals and institutions only, and that its report will be issued almost immediately.

### "HOW TO USE THE NEW JOURNALISM."

The March number of *Progress*, the Journal of the Great Northern Central Hospital, contains a long extract from the recent leading article in The Hospital under the above title. In an interesting comment, alluding to the subjects of general hospital interest which have been treated in *Progress*, its editor says: "The Hospital leader brought requests for *Progress* from various hospitals throughout the United Kingdom. We shall be pleased to receive (for insertion in our Journal) contributions appertaining to the voluntary hospital system." We welcome this endorsement of our proposal for the exchange of ideas and of readers among hospital journals. If other papers will take the large view which has led *Progress* to publish articles, by contributors to other kindred journals, on general hospital problems, institutional newspapers will exert twice their present influence by combining their several readers and, without sacrificing a jot of their present independence, will weld into a common body the supporters at present attached mainly to their local hospital alone.

### A STATESMANLIKE PROPOSAL.

Sir John Barnsley, chairman of the general committee of the Queen's Hospital, Birmingham, expressed definite views upon the future of the voluntary system at the recent meeting of the hospital's supporters. After reporting a debt of £20,500, and paying a deserved tribute to the workers, he went on to urge that there are many people outside the scope of the Saturday Fund whose support might be organised on kindred principles. He mentioned particularly shopkeepers, banks, stockbrokers and insurance companies. The latter probably will soon contribute to hospitals for the treatment which they now give to insured persons, and thus mend the radical defect of this unpopular measure, a defect exposed as soon as the Act was introduced. But the remaining classes mentioned by Sir John Barnsley await organisation on voluntary lines. We have urged this so often that we cannot but welcome another advocate; and since Birmingham and the provincial cities often give the lead to London in such matters, we hope that Sir John Barnsley will press his proposal, and see if the guild organisation which he recommends cannot be started. There is a Hospital Council in Birmingham, and such a body, with which all the hospitals of the city are in relation, would fully justify its existence if it would set such a guild upon its feet. To avoid confusion with the new guild lately started, some other name might be chosen. But the main point is to start such a fund for those beyond the scope of the Hospital Saturday movement. The initiator would be doing a work of great value, and Sir John may count on all the support which we can give.

### RED CROSS GRANTS FOR BUILDING.

It will be remembered that a large number of the Red Cross grants made through King Edward's Hospital Fund were conditional upon a similar amount being obtained by a hospital towards the improvement fund, which had secured the promise of a grant. Since this time, however, nearly a year ago, hospitals have been as a rule too busy collecting funds for maintenance to pay much attention to appeals on behalf of enlargement schemes, etc. The time-limit for claiming the Red Cross grant is drawing near, and most hospitals are making strenuous efforts at the eleventh hour to get sufficient support to the building fund to justify a claim for the Red Cross grant, which had been conditionally allocated. One of these is the London Temperance Hospital, which needs £1,500 for the Vezey-Strong Memorial Wing. The amount already subscribed is £4,500, and a corresponding sum has been paid over from Red Cross funds, but to secure the full grant the amount mentioned above must be forthcoming within the next few days. The proposed new wing is intended as a memorial of the thirty years' work given to the hospital by the late Sir T. Vezey-Strong.

### MENTAL HOSPITAL WORK IN SYRIA.

The London Committee of the Lebanon Hospital for Mental Diseases at Asfuriyeh, near Beirut, Syria, is anxious that the institution's work shall be better known if only that it may be able to pay its way; the first necessity is to pay off a debt of about £800. It is hoped to hold lantern lectures and drawing-room meetings. These are well enough, but why does not the Committee from its office in Queen Victoria Street draft a well-written account of the hospital's work, the difficulties of that work in Syria, the prejudices of the patients, and accompany it with illustrations? If such an account appeared first in hospital newspapers, and were really well done, a less technical article with other photographs would probably find a welcome in the illustrated weeklies. We are always glad of first-hand information of the remoter hospitals abroad.

### A NEW DUTY FOR IRISH HOSPITAL SECRETARIES.

The appalling condition of Ireland has added to the public duties exacted from hospital secretaries in that unhappy country. Under the Restoration of Order in Ireland Act hospital secretaries have been required daily to inform the military authorities of the names and particulars of persons treated for gunshot and similar wounds. Eleven Dublin hospitals, under the Act of 1856, receive State grants, and are supervised by a State Board of Control, which issues annually a report to Parliament. One of these hospitals, Dr. Steeven's, received in 1918 eighty-one such cases. The new duty excites criticism because it has occurred at the time when the request to increase the State grant, because of the fall in the value of money, was rebuffed.

### ANOTHER LOSS TO CLAYTON HOSPITAL.

We regret to record the death of Mr. Percy Tew, J.P., D.L., President for over forty-one years of the Clayton Hospital, Wakefield, which took place at his Sussex residence on March 24. Mr. Tew's services to and interest in the Clayton Hospital during the whole of his long presidency were unstinted, and even up to the last, though in his eighty-first year, he took an active part in the work and retained his wonderful grasp of the details of hospital management. He was buried at Wakefield, and a special and largely attended service was held in the Cathedral where less than a month ago a similar service was held for the late matron of Clayton Hospital, Miss Pressland, who had held office for nineteen years.

### BELGRADE CHILDREN'S HOSPITAL.

Dr. Katherine Macphail, who went to Serbia with the Scottish Women's Hospital, of which Dr. Elsie Inglis was the head, has now established a children's hospital in Belgrade. At present the hospital is in temporary quarters only, where she has a small British staff, helped out by some Slav sisters, but in April she hopes to move into a permanent hospital with 100 beds, the estimated cost of which is £5,000. There is, in addition, a summer pavilion at Topchider, in the Crown Prince's deer forest, with thirty beds, and a villa near Ragusa for a sanatorium for tuberculosis. Dr. Macphail hopes not only to perfect this, the first children's hospital in the country, but to encourage the foundation of others and to train native nurses to work in them. The Government is sympathetic, and provides soldier servants to help in the work. A local committee supplies a great part of the necessary food, and Dr. Macphail has the support of the Ministry of Child Welfare. As there are in Old Serbia alone half-a-million orphans, it is obvious that there is need for every form of child welfare, and Dr. Macphail's enterprise will do much to help the recovery of our sorely-tried Slav ally.

### WHAT DOES A PAYING PATIENT COST?

Mr. R. Pickles, Wigan Board of Guardians, lately made a useful speech in which he urged the advisability of admitting paying patients into the infirmary so far as there is fit room for them. But, because of the Ministerial call for economy, he remarked that no expense must fall upon the rates. Other guardians are admitting such patients at a fee of three guineas a week. What, he asked, is the real cost of a private patient in an infirmary? The information, especially if it varies, would be interesting.

